# An uncommon presentation for a severe invasive infection due to methicillin-resistant *Staphylococcus aureus *clone USA300 in Italy: a case report

**DOI:** 10.1186/1476-0711-7-11

**Published:** 2008-04-30

**Authors:** Piero Valentini, Gabriella Parisi, Monica Monaco, Francesca Crea, Teresa Spanu, Orazio Ranno, Mirella Tronci, Annalisa Pantosti

**Affiliations:** 1 Istituto di Clinica Pediatrica, Università Cattolica del Sacro Cuore, Largo Agostino Gemelli 8, 00168 Rome, Italy; 2 Dipartimento dei Servizi, Azienda Ospedaliera San Camillo Forlanini, Circonvallazione Gianicolense 87, 00152 Rome, Italy; 3 Dipartimento di Malattie Infettive, Parassitarie ed Immunomediate, Istituto Superiore di Sanità, Viale Regina Elena 299, 00161 Rome, Italy; 4 Istituto di Microbiologia, Università Cattolica del Sacro Cuore, Largo Agostino Gemelli 8, 00168 Rome, Italy

## Abstract

**Background:**

Methicillin resistant *Staphylococcus aureus *(MRSA) has been considered for many years a typical nosocomial pathogen. Recently MRSA has emerged as a frequent cause of infections in the community. More commonly, community-acquired (CA)-MRSA is a cause of infections of the skin and soft-tissues, but life-threatening infections such as necrotizing pneumonia and sepsis can occasionally occur.

**Case presentation:**

This report describes an uncommon presentation of invasive CA-MRSA infection in an adolescent without known risk factors. The presentation was typical for bacterial meningitis, but the clinical findings also revealed necrotizing pneumonia. Following the development of deep venous thrombosis, the presence of an inherited trombophilic defect (factor V Leiden) was detected. The patient was successfully treated with an antibiotic combination including linezolid and with anticoagulant therapy. CA-MRSA was isolated from both cerebrospinal fluid and blood. The isolates were resistant to oxacillin and other beta-lactam antibiotics and susceptible to the other antibiotics tested including erythromycin. Molecular typing revealed that the strains contained the Panton-Valentine leukocidin genes and type IV SCC*mec*, and were ST8, *spa *type t008, and *agr *type 1. This genetic background is identical to that of the USA300 clone.

**Conclusion:**

This report highlights that meningitis can be a new serious presentation of CA-MRSA infection. CA-MRSA strains with the genetic background of the USA300 clone are circulating in Italy and are able to cause severe infections.

## Background

Although considered a typical nosocomial pathogen for a long time, methicillin resistant *Staphylococcus aureus *(MRSA) has recently emerged as a cause of infections in the community. Most commonly, community-acquired (CA)-MRSA causes skin and soft-tissue infections [1,2] while severe and life-threatening infections such as necrotizing pneumonia [3], necrotizing fasciitis [4] and severe sepsis [5] represent rare cases. CA-MRSA strains are globally spread but their prevalence and distribution vary from country to country [6]. In the United States, CA-MRSA isolates have become major community pathogens [1] and are starting to be introduced into the health-care system [7]. In that country, most of the recent CA-MRSA isolates belong to a single clone, that can be identified on the basis of Pulsed Field Gel Electrophoresis (PFGE) profile and other genotyping characteristics and is designed USA300 [2]. In Europe, the prevalence of infections due to CA-MRSA appears to be lower than in the United States, although recent reports highlight that these infections are on the rise. In a French hospital the incidence of skin and soft tissue infections due to CA-MRSA increased from 0% in 2000 to 6.8% in 2003 [8]. In Copenhagen the number of MRSA isolates, mainly obtained from community-acquired skin infections, doubled in less than 1 year, from 2003 to 2004) [9]. Differently from the situation in the United States where one clone predominates, CA-MRSA clones circulating in Europe are more diverse and heterogeneous [6].

In CA-MRSA isolates *mec*, the gene coding for methicillin resistance, is usually contained in the type IV variant of the genetic element staphylococcal chromosomal cassette (SCC)*mec*, that is smaller and probably more mobile than the other SCC*mec *elements [10]. CA-MRSA often contains the Panton-Valentine leukocidin (PVL), a toxin endowed with the unique ability to kill leukocytes [11]. PVL was initially associated with skin infections [12] and recently has been shown to play an essential role in the pathogenesis of necrotizing pneumonia [13].

We describe here an uncommon presentation of invasive CA-MRSA infection, with meningitis, bacteremia and necrotizing pneumonia in association with deep vein thrombosis, in a previously healthy adolescent.

## Case presentation

**Figure 1:**
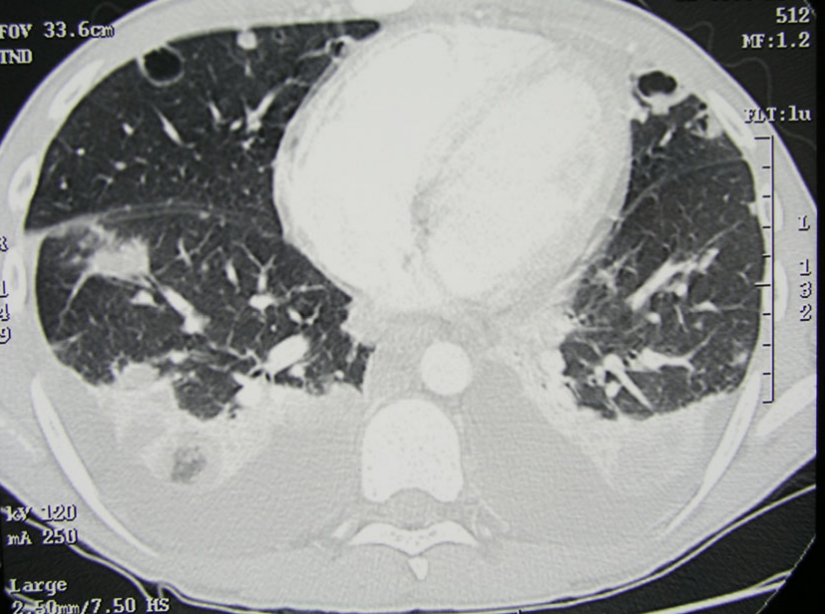
**Chest CT of a patient with necrotizing pneumonia caused by CA-MRSA USA300 genotype**. The CT scan, obtained on hospital day 5, shows multiple nodular lesions, some with a central cavitation, and bilateral pleural effusion.

A 15-year-old boy presented to the emergency department with a 4-day history of headache, fever, and lumbar pain and 1-day history of vomiting. His past clinical history was unremarkable, with the exception of mild asthma during childhood. Initial examination showed: temperature 40°C, heart rate 120 beats/min, respiratory rate 30 breaths/min, blood pressure 120/78 mmHg, mild disorientation (Glasgow Coma Scale 14) and meningeal signs. A small infiltrating skin lesion, partly crusted and partly purulent, was present on the back. The boy had been on a trekking vacation in Sicily and the lesion had been reportedly caused by the backpack on the bare skin. A chest radiogram showed bilateral multiple nodular infiltrates. Ultrasound examination of the abdomen revealed moderate enlargement of the liver and the spleen. A brain CT scan was normal. Analysis of blood samples at admission revealed a white blood cell count of 10,300 cells/mm^3 ^with 86% polymorphonuclear leukocytes. Arterial blood gas analysis showed moderate hypoxemia (Pa_O2 _of 73 mm Hg on room air). Blood cultures and a lumbar puncture were performed. Cerebrospinal fluid (CSF) examination revealed several red blood cells and a leukocyte count of 600 cells/ml, a protein level of 392 mg/dL, and a normal glucose level. Antibiotic therapy with clarithromycin and ceftriaxone was initiated and the patient was transferred to the Pediatric Intensive Care Unit (PICU) of another hospital, where his therapy was changed to ampicillin, doxicycline, ceftriaxone and acyclovir. Laboratories studies revealed a white blood cell count of 8,700 cells/mm^3 ^with 84.8% polymorphonuclear leukocytes, platelet 130,000/mm^3^, C-reactive protein 187 mg/l (normal value < 3 mg/l), prothrombin time 20.90 sec, activated partial thromboplastin time 42 sec, international normalized ratio 1.57, fibrinogen 1060 mg/dL (normal range 200–400 mg/dL), antithrombin III 39.8% (normal range 70–120%), D-dimer > 4500 ng/ml (normal value < 278 ng/ml). The patient did not require mechanical ventilation and after 18 hours was discharged from the PICU and readmitted to the pediatric ward in stable conditions, conscious and still febrile to 40°C. Blood and CSF cultures from admission grew MRSA. Ampicillin was discontinued and vancomycin was added. Since fever persisted unabated, on day 5 the antibiotic therapy was switched to teicoplanin (400 mg iv twice a day), linezolid (600 mg iv twice a day) and rifampicin (600 mg iv once a day). A heart ultrasound revealed no valvular vegetations. A CT scan showed bilateral lung cavitary lesions suggesting multiple abscesses, bilateral pleural effusion (Fig. 1), thrombosis of the inferior vena cava below the renal veins, and of both iliac veins. Low molecular weight heparin was started. The patient demonstrated gradual clinical improvement, with slow normalization of temperature and C-reactive protein concentration. CT scan showed reduction of the nodular lesions and of the pleural effusion. Abdomen CT scan and echo-colour Doppler revealed resolution of the venous thrombosis. The patient completed a 5-week course of teicoplanin, rifampicin, and linezolid and was discharged home at day 40 in good general conditions. Anticoagulation was continued for a total of 6 months.

Because of the unusual severity of the infection, underlying immunological or hematological defects were investigated. Quantitative immunoglobulins and subtypes, lymphocyte subtypes, and phagocyte function were normal. The presence of heterozygous factor V Leiden and prothrombin gene mutation (G20210A) was revealed.

### Phenotypic and genotypic characterizations of MRSA

*S. aureus *was identified by standard microbiological methods. Antibiotic susceptibility testing was performed by BD Phoenix™ Automated Microbiology System (Beckton Dickinson) and confirmed by E-test (AB Biodisk, Solna, Sweden). The species identification and the methicillin resistance status were confirmed by a duplex PCR targeting the genes *nuc *and *mec *[14]. MRSA grew from the blood and CSF cultures obtained at admission and from a second blood culture performed after 48 h, before the institution of vancomycin therapy. The isolates were resistant to oxacillin but susceptible to non-beta-lactam antibiotics including erythromycin, clindamycin, ciprofloxacin, tetracycline, rifampicin, gentamicin, vancomycin, teicoplanin and linezolid. By E-test, the MIC for vancomycin was 2 μg/ml. Further characterization of the isolates was obtained by PCR assays and included determination of the SCC*mec *type, *agr *group, and presence of the PVL genes *lukS-PV *and *lukF-PV *[14]. Genotyping of the strains was obtained by PFGE, Multilocus Sequence Typing and by sequencing the polymorphic region of protein A (*spa *typing) as previously described [14]. The isolates from blood and CSF were identical by PFGE, and carried SCC*mec *type IV and the genes coding for PVL. Molecular typing showed that the strains were *spa *type t008, ST8 and a*gr *type 1, characteristics shared by the USA300 CA-MRSA clone.

## Conclusion

The development of a serious infection in a young person without predisposing conditions is a typical feature of CA-MRSA infections. However, to our knowledge, this is the first described case of CA-MRSA infection presenting with meningitis in an adolescent. The isolation of CA-MRSA from CSF is not common. CA-MRSA was isolated from the CSF of a 16-month-old child who died from fulminant sepsis. She had multiple abscesses in several organs, including the brain [15]. A case of CA-MRSA meningitis in an adult subject was mentioned in a study from Hong-Kong, but apart from the unfavorable outcome, clinical data are lacking [16]. Another fatal case recently reported, was that of a woman with a history of furunculosis and drug addiction, who developed a brain abscess due to CA-MRSA [17]. In our patient, meningeal symptoms were predominant at presentation, and the respiratory symptoms remained moderate in spite of the large involvement of the lungs as demonstrated by CT scan. The patient did not present airways hemorrhage, nor leukopenia, both factors being associated with fatal outcome in necrotizing pneumonia [18]. The skin infection on the patient's back might have represented the initial source for hematogenous spread of CA-MRSA, although microbiological examination of the purulent secretion was not performed.

Bacteremia due to typical CA-MRSA containing the PVL genes is rare but not exceptional, since typical CA-MRSA can be isolated from blood in the course of severe sepsis [5] or in other serious CA-MRSA infections in children and adolescents [19,20]. In an unselected patient population, bloodstream infections due to PVL-positive CA-MRSA are very rare in Europe, as shown in a recent study from England [21] that included mainly isolates from elderly patients. In the USA, an increasing number of bloodstream infections in hospitalized patients is due to typical PVL-positive CA-MRSA [22], probably reflecting the introduction of the USA300 clone in the health-care setting in that country.

Another remarkable feature of this case is the association of the invasive CA-MRSA infection with deep venous thrombosis (DVT). DVT has been increasingly observed in children in association with osteomyelitis due to *S. aureus *containing the PVL genes [23]. Since the presence of PVL seems to be associated with greater systemic and local inflammation [20], it has been hypothesized that PVL could contribute substantially to the development of DVT. The expression "PVL syndrome" has been proposed to indicate a multifocal infection including osteomyelitis, skin infections, pneumonia, and DVT, due to PVL-positive *S. aureus *[24]. However, the association between PVL and DVT has been only observed in acute osteomyelitis and a causative role for PVL is unproven. In our patient, the CA-MRSA infection has uncovered inherited trombophilic defects, the factor V Leiden and the prothrombin gene mutation, that have determined the development of DVT, with or without the contribution of PVL. On the other hand, the presence of heterozygous factor V Leiden is known to confer protection in the septic state and decrease sepsis-related mortality [25], therefore it can have contributed to the favorable outcome of this case.

The CA-MRSA strain involved had genotypic characteristic (*agr *type 1, ST8, t008) of the USA300 clone that has become the predominant CA-MRSA clone in the USA. In Europe, the most common clone is the "European clone", characterized by *agr *type 3 and ST80 [26], and typically resistant to fusidic acid [27]. Recently, strains with the USA300 genotype are starting to be isolated with increasing frequency in various European countries [6]. In Germany, CA-MRSA isolates with the characteristics of the USA300 clone have become the second most frequent, after the ST80 isolates [28]. In Italy, a strain with the genotyping characteristics of the USA300 clone was recently recognized as the cause of a serious skin infection in a child [29].

The empirical therapy started for meningitis and community acquired pneumonia in this patient, that included ceftriaxone and clarithromycin, is ineffective against MRSA. In addition, ceftriaxone and beta-lactam antibiotics in general have been shown to increase the expression of PVL by *S. aureus *[30], thus potentially worsening the disease. When the results of the antibiotic susceptibility tests were available, vancomycin was started. However, since the vancomycin MIC of the isolate was at the breakpoint for susceptibility (2 μg/ml) and the patient's condition remained critical, a different antibiotic combination including linezolid was started. Linezolid was chosen because of its high antistaphylococcal activity, and its excellent CSF and lung tissue penetration. As in the previously described case of necrotizing pneumonia [14], linezolid therapy likely contributed to the favorable resolution of the CA-MRSA infection.

The antibiotic treatment of this patient raises several questions and reflects the lack of evidence-based data to guide the choice for the treatment of serious CA-MRSA infections. Vancomycin was used as the first-line treatment since it is effective against MRSA, and has been widely used in the past. Although vancomycin penetration in CSF is low, concentrations of 6–11 μg/ml are usually reached with inflamed meninges [31] and vancomycin is currently recommended in the antibiotic combination for the empirical treatment of bacterial meningitis [32]. The patient had also pneumonia and it is known that the levels reached by vancomycin in the lung compartment can be variable and insufficient for a bactericidal effect [31]. Poor penetration into the infected sites was a particularly critical issue in this case, since the MIC for vancomycin of the isolates was 2 μg/ml, that is still in the susceptibility range but close to the breakpoint. Clinical failures have been reported in serious infections due to MRSA with reduced susceptibility to vancomycin [33].

All the above conditions might have determined the lack of clinical improvement of the patient. Therefore, a new antibiotic combination was attempted, including teicoplanin, rifampicin and linezolid. Although there are few clinical studies relating to the use of teicoplanin in meningitis, in a rabbit model of MRSA meningitis the antibacterial activity of vancomycin and teicoplanin was shown to be similar [34]. Recent studies have shown the efficacy of linezolid in the treatment of patients with central nervous system infections [35] and pneumonia [36], its efficacy being at least equivalent and in some instances superior, to that of vancomycin. The drug combination was chosen based on the *in vitro *susceptibilities of the isolates but no adjunctive *in vitro *tests were performed to show whether the combination had synergistic, additive or antagonistic activity. There are no published clinical studies with this antibiotic combination to support the choice.

This case adds a new clinical presentation to CA-MRSA infections and highlights the problems encountered in the choice of the therapy of serious community-acquired infections in the CA-MRSA era.

## Competing interests

The authors declare that they have no competing interests.

## Authors' contributions

PV participated in the design of the study and helped drafting the manuscript. GP conceived of the study and performed the antibiotic susceptibility of the bacterial isolates. MM carried out the molecular typing of the isolates. FC collected and analyzed the clinical data of the patient. TS contributed to the identification and antibiotic susceptibility of the isolates. OR produced the hematological data of the patient and participated in the coordination of the study. MT participated in the design of the study. AP coordinated the study and drafted the manuscript. All authors read and approved the final manuscript.

## Consent

Written informed consent was obtained from the patient's parent for publication of this case report and of the accompanying image. A copy of the written consent is available for review by the Editor-in-Chief of this journal.
